# Research Care and Teaching Care Research in the Faculty of Nursing at Universidad de Antioquia. Testimonies and Legacies in 70 years of Institutional Life, 1950-2020

**DOI:** 10.17533/udea.iee.v38n3e03

**Published:** 2020-11-11

**Authors:** Elvigia María Posada Vera, Beatriz Elena Arias López

**Affiliations:** 1 Nurse, Ph.D. Professor. Email: emaria.posada@udea.edu.co emaria.posada@udea.edu.co; 2 Nurse, Ph.D. Professor. Profesora. Email: beatriz.arias@udea.edu.co beatriz.arias@udea.edu.co; 3 Facultad de Enfermería, Universidad de Antioquia, Medellín, Colombia. Universidad de Antioquia Universidad de Antioquia Medellín Colombia

**Keywords:** nursing research, education, nursing, nursing care, research support as topic, investigación en enfermería, educación en enfermería, atención de enfermería, apoyo a la investigación, apoyo a la investigación como asunto, pesquisa em enfermagem, educação em enfermagem, cuidados de enfermagem, apoio à pesquisa como assunto

## Abstract

The development of care research and its teaching in the Faculty of Nursing at Universidad de Antioquia cannot be understood outside the context in which it emerges and from the trajectory of its leading players. This is how this article will present a synthesis of the future of research, with its principal milestones and events in institutional history, in dialogue with the trajectories of four teaching nurses, protagonists of key moments in said history and living testimony of 70 years of institutional life. This panorama seeks to constitute a referent for strategic reflection, which inspires new generations to meet the research challenges and teach care research.

## Resumen

El desarrollo de la investigación del cuidado y de su enseñanza en la Facultad de Enfermería de la Universidad de Antioquia no puede ser entendido por fuera del contexto en que emerge y de la trayectoria de sus protagonistas. En este artículo se presentará una síntesis del devenir de la investigación, con sus principales hitos y acontecimientos en la historia institucional, en diálogo con las trayectorias de cuatro enfermeras docentes, protagonistas de momentos claves de dicha historia y testimonio vivo de 70 años de vida institucional. Este panorama pretende constituir un referente para la reflexión estratégica que inspire a las nuevas generaciones para responder a los retos de la investigación y la enseñanza de la investigación del cuidado.

## Resumo

O desenvolvimento da pesquisa do cuidado e seu ensino na Faculdade de Enfermagem da Universidade de Antioquia não podem ser compreendidos fora do contexto em que se inserem e da trajetória de seus protagonistas. É assim que este artigo apresentará uma síntese da evolução da pesquisa, com seus principais marcos e acontecimentos na história institucional, em diálogo com as trajetórias de quatro enfermeiras professoras, protagonistas de momentos chaves de dita história e testemunho vivo de 70 anos de vida institucional. Este panorama pretende constituir um ponto de referência para a reflexão estratégica, que inspire as novas gerações a responder aos desafios da investigação e do ensino da investigação em cuidados.

## Introduction

As indicated by Fawcett,(1) it would seem that nursing research has operated similarly in different parts of the world, having as foundational milestone the pioneering works by Florence Nightingale in 1850. With asynchronies characteristic of the global north and south, the first undertook research works and formation processes since the 1920s, with strong tendency to conduct studies related with nursing education, which were then energized around themes related with the health experiences of people and communities during the 1970s, seeking to create an evidence base to support care practices,(2) which has been maintained to current times.

The need to research the nursing practice managed to consolidate itself in the 1980s, with an important increase in the number of nurses carrying out research projects and discussions in theoretical and contextual themes that led to understanding nursing as art and as science.(3) This trend is strongly linked to the dynamics of nurse formation centers, which particularly in Colombia, became a legacy of the Faculty of Nursing at Universidad de Antioquia. During its 70 years, this study center, from 1950 to 2020, the institutional trajectory is intersected with biographical accounts of nurses who constructed the day to day, through questions and diverse modes of doing research and teaching the craft of researching, positioning and generating knowledge about nursing and care, with regional, national, and international reach.

This article will tour the milestones and events of the institutional life in a framework with personal testimonies, whose purpose is to open and propitiate a setting for intergenerational dialogue, an approach to the exchange of memories, a wager and concern for the “heritage” to new generations. These memories, more than a nostalgic remembrance, seek to position a living speech of dialogic nature,(4) to recognize and emphasize diversity, differences, conflicts, dissents, and struggles, as well the lessons, supports, and agreements, synthesized in every memory.

## Institutional milestones and trajectories

Although since early 20^th^ century, the need to form nurses duly trained in the city of Medellín, Colombia was evident, the initial training processes were not conducted with the purpose of leading to any degree and, rather, responded to specific needs, which were supported by professors from the Faculty of Medicine at Universidad de Antioquia.(5) It was only by the middle of the century, as response to recommendations by North American medical missions, who visited the country between 1944 and 1948, that the importance was acknowledged of “creating schools of nursing, as response to needs felt by society”,(6) with the religious community of the Sisters of the Presentation in Medellín assuming and materializing this idea through the foundation, in March 1950, of a School of Nursing, with the attendance of 11 members from the religious community.

Although at the beginning, the nascent School of Nursing was destined for the formation of people from the religious community, the directives at Universidad de Antioquia requested considering also secular individuals and jointly create a school of professional nurses with capacity to provide health services based on scientific knowledge and backed by this institution. This is how in September 1950 began the School of Nursing at Universidad de Antioquia, under the direction of the Sisters of the Community of the Presentation.(3)

At this time, learning was marked by a technical and instrumental logic, with strong influence from the biomedical model. In the Colombian context, debates had not been undertaken that in the Anglo-Saxon world already claimed recognition of nursing as a scientific discipline, independent and autonomous. During this period, the precariousness in access to scientific production characteristic of the profession, limited significantly early access to areas as important as research, and to progress in the disciplinary discourse. Even up to the 1960s, although the importance of strengthening training in areas, like administration and teaching, was emphasized, research as such did not have a preponderant place.(7) Very incipiently in 1957, the Advisory Council that presided over the then School of Nursing approved the obligation of an academic work that approached what was considered a degree thesis as condition for the students to receive their degree. Orientation and approval of said works was in charge of the School’s teaching physicians.(3)

Thereafter, new debates in the formation model led in 1965 to the establishment of a complementary cycle to the basic program of nursing formation, which permitted opting for the Degree in Nursing. At that time, the influence of the hygienist current and the preventive model of public health permitted encouraging formation in epidemiology and biostatistics, considered important tools for research work. It also included formation contents related with the methodology of social research, evaluation techniques, communications, among others, which directly or indirectly provided nursing students tools to conduct research.(6)

This is how after 15 years from the creation of the Nursing program at Universidad de Antioquia, positivist research was added to the formation of professionals, marking a first milestone in the institutional and professional trajectory in research, which kept a preponderant place as hegemonic trend until the 1980s. This tradition collected the imprint of Nightingale and her renowned publication Notes on Nursing, which aimed for measurable and concrete knowledge related with issues of care.(8)

Although the Degree in Nursing was approved in 1965 and perfected in 1967, it favored access to graduate studies, like specializations and Master’s studies, only a few professors sought access to them during the early 1070s, especially in epidemiology, with which they became a privileged group, carriers of specialized knowledge and special training, which as of that moment insinuated the tension that remains to today, of dedication to research or to teaching in research for “closed groups”, which leaves out an important number of teaching faculty, generating relations of power and resistance against the social status that accompanies the figure of researcher. Paradoxically, the scarcity of professors with graduate formation, not only established the idea of this species of “research elite”, but a certain aversion to research, considering that this was an activity of little significance for the development of nursing, whose focus was indissolubly linked to the care practice. That is, the idea of research as motor for disciplinary development, which was already consensual in other geographies, was a matter questioned in our context, which still had to deal with bias established on the distance among research, nursing, and the care practices, besides the political and ethical debates around the research practice.(3)

Later in the decade, multiple debates were stirred and grew, claiming the need to transcend the clinical, individual, and biomedical vision to approach an integral vision of the people and of the health-disease process, recognizing the importance of the approach among disciplines.(9) This understanding stirred great interest in the study of the existing relation between disease and society, which led to the creation of the movement of social medicine in Latin America.(7) In Colombia, this movement began growing between the 1970s and 1980s, around specific programs in some universities, including Universidad de Antioquia. Professors and students from the health area, with an important representation from the School of Nursing, addressed the country’s sociopolitical problematics, demanding greater critical conscience and a broader and humanistic vision of health and of the social context, which entered into conflict with the biomedical and positivist approach strongly positioned in the School.(3)

All this raised changes in the formation and the nurses’ practice, recognizing that the solution of problems was necessarily the competence of intersectoral actions, when facing the health-disease phenomenon as a collective, historical, and social reality and, hence, complex, which is why interdisciplinary team work emerged as an unavoidable response.(3) With this new orientation, a second milestone was positioned in the research trajectory of the School of Nursing at that moment, establishing the theoretical and epistemological debate around the methodological monism and giving rise to interpretative research, an approach consolidated during the 1980s and which became a seal of institutional and professional recognition after the 1990s, with permanence to date. In the 1980s, the purposes of research development materialized in the Research Center of the Faculty of Nursing, which began in August 1981 and in the conformation of academic and work groups, which would later migrate to the research groups now in the Faculty; as well as with the offer of graduate programs that began during this decade, which grew and remain until now.

On par with these changes, a new philosophy was proposed in the formation of nurses in the early 1980s, which besides claiming greater social and political awareness, also advocates for autonomous, purposeful, and reflective nursing upon social, institutional, and disciplinary needs.(3) At that given moment, the Superior Council at Universidad de Antioquia approved the project that transformed the school into Faculty of Nursing and which established a professional profile that explicitly declares the importance of: *a close relation with the theoretical, methodological, and technical elements of social and epidemiological research to analyze the relation of health problems with the economic, sociopolitical, and demographic structures, guiding the search for real solutions to problems not only from biological alterations, but from the expression of multiple determinations of the social structure*.(6)

By this time, a public declaration was instituted committed with the formation of nurses for quality performance in their four basic functions: care, teaching, administrative, and research in the clinical and community environments.

We could, then, state that, after 30 years of existence of the Faculty of Nursing at Universidad de Antioquia, research had managed to position itself and secure a place of legitimacy as guarantor of scientific and disciplinary development, under a vision of approaches in tension, which were mobilized and emerged in the classrooms and in the teaching-learning process. Precisely during this decade, in 1983, the journal Research and Nursing Education, founded on the recognition research had gained in the faculty and the scientific production derived from it, which merited the creation of a dissemination instrument. In spite of the gains indicated, it was a process with stumbling blocks, given that nursing was still seen by most people as a technical profession dedicated only to practice, with total dependence on the medical discourse. The journal managers skewed the generalized idea that research was an unknown and foreign exercise for nursing; making a balance of research being conducted since the 1970s, which mostly had not been published, given the scarcity of dissemination media that allowed these works to be made visible; an inventory published in the first number of *Research and Nursing Education* in 1983.(3) It must be indicated that this was the third journal of scientific dissemination in nursing in Colombia, which had been anteceded by the *ANEC* journal published by the National Association of Nurses of Colombia since 1966 and *Advances in Nursing* at Universidad Nacional, published since 1982.

In parallel manner, with the initial work of the journal, its pioneers also traced the first lines of nursing research, a totally novel theme because it was the first time this was talked about in the country, and specifically of lines in nursing, which was of interest both in and out of the university, making visible in an important way the faculty throughout the country. The product of the reflections on the lines of research was published in the second number of the journal *Research and Nursing Education* in March 1984, when neither the university or COLCIENCIAS (now MinCiencias) referred to these forms of organization of the research activity.(6)

As mentioned, synchronously and complementary to these initiatives, the Research Center of the Faculty of Nursing was also created, which was founded to train members from the academic unit on research, manage research, and disseminate knowledge. From this center, arduous work was undertaken on the conformation and positioning of the lines, managing to consolidate several of them as care for adults and children in critical state of health; health of ethnic groups; the nursing practice in the social context; gender and health; sexuality and the clinic; end-stage care, death and palliative care; and interest groups around the dynamics of health professionals and reforms to the sector.(10) All this progress positioned the Faculty as a reference center for internal and external training and advice to nurses from health institutions and also to the Colombian Association of Faculties of Nursing (ACOFAEN, for the term in Spanish) on the preparation and execution of academic events.(6)

Amid these dynamics, the 1990s emerged with strong reforms of global nature, which liberalized economies and imposed important reforms, especially in Latin American countries. Paradoxically, said decade left a constitutional reform that sought the recognition of a diverse and inclusive country, while also bringing the reform to the social security system in which the privatizing neoliberal ideas of social guarantees were embodied. Health and education began to take the course of the market, under the premises of free enterprise and reduction of the role of the State, which also marked the course of research, not only in its investment and funding priorities, but in the same logics of social function of knowledge, which for many authors takes shape in what has been called cognitive capitalism in the 21^st^ century, where the generation of critical thinking is also trapped by the logics of commodification and its formats of creation and dissemination of knowledge.(11)

Within the Faculty of Nursing at Universidad de Antioquia, the 1990s was a period of great and profound movements, characterized by great deliberations and understandings on the object of nursing reflection and practice, which, with great conceptual maturity, made important contributions to the academic production of the discipline in the country. In this regard, María Consuelo Castrillón (12) states that: *During this period “care” was identified as a historical function of nursing professionals, a function that has had diverse modalities and contents, and whose study must contribute to enriching the conceptual and action field*. (p. 24-25)

In terms of research, emphasis was made on the need to develop projects with other disciplines and, thus, generate feedback of technical-scientific knowledge with regional and national communities, and training was significantly encouraged in qualitative research. It was also, starting this decade, when the opening of the Master’s in Collective Health, which inaugurated research formation as a Faculty project. Management of this program, with participation by many teaching nurses who led other, already mentioned, projects related with the development of research in the Faculty of Nursing began by problematizing single-disciplinary training and some fields that were in the academic scene at the moment, as was the case of family health and community health. Finally, it was opted for collective health; considering this a novel and encompassing field, which in epistemological terms installed an interdisciplinary intention, in methodological terms proposed a diversity of comprehensive approximations, and in praxeological terms privileged a sociopolitical approach to comprehend and intervene the health-disease-care-death process.(13)

At the dawn of the 21^st^ century, research in the Faculty of Nursing had opted for important projects; on one side, a research center operating uninterruptedly for 20 years, five active research groups, a scientific journal for the dissemination of knowledge in nursing, development of some editorial projects, and projection and implementation of a solid graduate studies offer for formation in research, not only for nursing professionals, but for professionals from other disciplines. The first decade began with the approval of the Master’s in Nursing and closed with the approval of the Doctorate in Nursing.

Currently, closing the second decade, we encounter a complex panorama regarding research: the “false dilemma” among the methods,(14) the scientist status and “elitist” circuits of knowledge, the preponderance of cognitive capitalism, and the biotechnological challenges, besides a full context of demands derived from an epidemiological profile in transition of demographic transformations and of great social and environmental problems, which place at odds the local with the global, are only some of the challenges we must face. On par, now, unlike 15 years ago, research is part of the university dynamics of professors and students from the Academic Unit, that in a naturalized way circulates through the daily life of its different spaces and settings, with important qualification of the teaching staff on research, bearing in mind that 95% have studies at master’s and/or doctorate levels. These new generations today must respond to the challenges indicated and others related with the transit of mono disciplinary to inter and trans disciplinary studies (15) that integrate solid theoretical argument against care, which articulate care practices as source of knowledge and which consider the management of the results of nursing research, to bridge the gap between theory and practice of care.(16)

## Personal milestones and trajectories

The institutional path, previously mentioned, comes alive in the trajectory of teaching nurses, who in their reports bring memory to life. During the Research Days of the Faculty of Nursing in 2020, four nurses, professors and researchers participated, who have been part of the developments presented.(17) From the point of view of their biographical trajectories, they highlight how the formative research processes in undergraduate studies, included in the national curricula since the early years of the 1970s, were decisive in their professional and disciplinary development, given that these permitted understanding the social importance of research and, as stated by María Consuelo Castrillón “finding a place in the world”.(17)

The biographical reports indicate that these were the first incursions through formative research, which led them to seek research formation processes through undergraduate studies, in and out of the country, a strength with which then arrived to echo on the curricular and research mobilizations that took place during the 1970s and 1990s in the Faculty of Nursing. Added to the progress that in terms of research had been obtained by including courses in the study plan, motivation, and training of professors and students was the involvement of some professors with formation principally in master’s in epidemiology, public health, and education, who trained other professors and nurses in formation, comprising a solid, recognized, and highly respected academic group that gave national and international visibility to the Faculty of Nursing at Universidad de Antioquia.

These formation processes were, in addition, accompanied by active participation in associations and academic groups that permitted them an encounter with peers and with professionals from other disciplines, important referents in the field of health and research in Latin America. These experiences ratify the importance of the collective to produce debates leading to new questions and exploration routes, where the interdisciplinary took and continues occupying a central spot; besides hinting at a line that today is much more forceful around the internationalization of research as a promising path to generate knowledge on care. This path also identified the importance of understanding and recognizing the disciplinary dimension to drive a nursing practice based on theoretical thought.

The testimonies by María Consuelo Castrillón Agudelo, Clara Inés Giraldo Molina, María Mercedes Arias Valencia, and Beatriz Elena Ospina Rave(17) account for the importance of spiraled routes that combine actions of researching, teaching, acting, and investigating inspired in the metaphor of “teaching from the swamp”, that is, from the place of the expert researcher who accompanies novel researchers, but also from the strategy of “teaching to research, investigating”, which permits consolidating a conscientious and critical attitude in the formation of researchers. For such, their testimony invites to consider the theoretical challenges that permit an approach to multiple authors, with their diverse tones and voices; the flexibility to recognize other paradigms and incursion into disciplines different from nursing, under the premise of epistemological insufficiency of the mono-disciplinary. The need to recognize epistemological interconnections emanates precisely from the complexity of human problems and care practices, from the certainty of the uncertain, and from the infinity and at the same time partiality of the questions and their responses.

## In closing

The history of the Faculty of Nursing at Universidad de Antioquia in its 70 years of institutional life, trajectory that began in 1950 and which takes place in a transition from vocational nursing to professional nursing, has positioned research as a fundamental pillar for its dynamics and transformations, amid tensions and debates that still continue within a context that still owes social recognition to the nursing discipline, as legitimate and autonomous field of knowledge and practice.

The distinct approaches to research have occurred as product of academic and social mobilizations that over the years have imposed different challenges and which are translated into curricular transformations, where the question for care research and teaching of care research have been present.

At its 15 years of existence, the Faculty of Nursing already included in its formation proposal research elements of positivist tendency, of great influence during the 1960s and 1970s, which, beyond the epistemological or methodological debates, permitted - temporarily - for nurses of the time to become sensitive to the social importance of research and strengthen their capacities and skills in the research practice.

Without a doubt, the 1980s consolidated the greatest debates, where the question for interdisciplinarity emerged with force, as well as the debate for methodological monism. As a result of that, sharp discussions for the moment, context, and discipline were undertaken in this study center, related with the sense of reality to which care was aimed, the forms of relationship between research nurses and the subjects of care, and the reach in the explicative and/or comprehensive horizon of the process. As of that moment, academic groups and lines of research were steadfastly established, which later gave rise to the five research groups that currently would gather nearly 80 teaching researchers.

From the formative point of view, since the 1970s until now, the Faculty of Nursing remains in favor of formative research for undergraduate students, which it enhances through different courses and seminars that permit their acquiring tools and skills to construct research questions and problems, design response paths, and execute their projects. Likewise, it has maintained, since the 1980s, an offer of research formation in master’s programs, consolidated during the first decade of this century with the implementation of the second Doctorate in Nursing in Colombia. In this sense, research continues claiming and occupying an articulating place for the discipline, now also associated to discussions for technological innovation, social appropriation, and transfer of knowledge, with all their vicissitudes. It is agreed that nursing research is essential for its development as profession and that it is in the production of knowledge where support bases are obtained to develop as science and as art.

The year 2020, denominated the Year of Nursing by the Advisory Council of the World Health Organization, where the profession receives public institutional support, is also the year in which, for the first time for many generations, a global pandemic produces unthinkable changes in individual and collective lives. Undoubtedly, these combined events, again reveal caring for life as a fundamental issue,(18) updating questions related with their ontological status and claiming epistemological and methodological alternatives upon its complexity. Although social recognition of nursing does not manage to fulfill the expectations of those in the profession, it is fundamental to insist on that path of “lights and shadows” of questions and infinite, although partial, answers to those where we are summoned by the research practice because therein is where care is strengthened.

Now more than ever, ethical and political challenges come forward, involving care research and teaching of care research, which urgently must address profoundly ontological issues related with the limitations of the humanist visions, the anthropocentric preeminence, and the urgent decentralization towards a socio-biocentric vision, which besides human life, recognizes also the need to care for non-human life forms.(19) If during the 1980s and 1990s the legacy of the discussions for the social determination of the health-disease-care-attention-death process (13) permitted energizing the research processes under an interdisciplinary vision; even today, these formats seem limited by the comprehension of care phenomena that in their complex order claim transdisciplinary logics and reflection on the ontological status of that life care is concerned with. For a Faculty of Nursing with 70 years of life, with a convincing historical legacy and qualification programs in the formation of researchers in Nursing, but - likewise - with consolidated processes of formative research, these and other challenges that are part of the uncertainty brought by the 21^st^ century, will be tracing factors in the following years of institutional life.
